# Efficacy of sildenafil and high-dose anakinra in an MIS-C patient with pulmonary vasculitis: A case report

**DOI:** 10.3389/fped.2022.1015617

**Published:** 2023-01-04

**Authors:** Francesco La Torre, Gerolmina Calabrese, Katia Signorile, Francesca Bizzoco, Carla Mastrorilli, Antonella Strippoli, Doriana Amato, Francesco Carella, Ugo Vairo, Paola Giordano, Leonardo Milella, Fabio Cardinale

**Affiliations:** ^1^ Department of Pediatrics, Giovanni XXIII Pediatric Hospital, University of Bari, Bari, Italy; ^2^ Pediatric Intensive Care Unit, Giovanni XXIII Pediatric Hospital, Bari, Italy; ^3^ Pediatric Cardiology Unit, Giovanni XXIII Pediatric Hospital, Bari, Italy; ^4^ Department of Interdisciplinary Medicine, Pediatric Section, University of Bari, Bari, Italy

**Keywords:** anakinra, children, COVID-19, MIS-C, multisystem inflammatory syndrome in children, pulmonary hypertension, pulmonary vasculitis, sildenafil

## Abstract

Multisystem inflammatory syndrome in children (MIS-C) is a newly identified clinical entity still not very well known in terms of epidemiology, pathogenesis, and long-term outcome. Pulmonary involvement with acute respiratory failure is an unusual life-threatening complication of MIS-C, often a reason for admission to the pediatric intensive care unit (PICU) and the use of mechanical ventilation. We present a case of a 7-year-old male patient, previously healthy, hospitalized for MIS-C, treated with intravenous immunoglobulins (IVIG), high dose methylprednisolone, and anakinra. After 2 days of the aforementioned therapy, the patient presented with hypoxia (SatO_2_: 85% in ambient air room) and breathing difficulties. A chest computed tomography (CT) scan showed the presence of multiple bilateral basal parenchymal thickening and small basal pleural effusion and an arterial blood gas analysis revealed severe hypoxia (PaO_2_/FiO_2_ ratio, 170 mmHg). Because of a worsening of respiratory distress, the patient was transferred to the PICU, where invasive mechanical ventilation and a continuous infusion of anakinra (12 mg/kg/day) were started. An echocardiogram was performed, which showed an increase in pulmonary pressure (40 mmHg) with normal heart ejection fraction (55%), and the hypothesis of pulmonary vasculitis involving the pulmonary arterioles was made. Therefore, therapy with sildenafil (0.15 mg/kg/day) was promptly set up, with an immediate improvement of the clinical picture of respiratory failure, reduction of pulmonary pressure (23 mmHg), and subsequent extubation at 36 h with a regular clinical course until discharge. As far as we know, our case represents the first report of pulmonary vasculitis in an MIS-C patient. The use of sildenafil and high-dose continuous anakinra may represent a rescue therapy in cases of MIS-C with pulmonary vasculitis or with difficulty in extubation, allowing a short-term hospitalization in intensive care and improving the long-term outcome in these patients.

## Introduction

Since the first phase of the COVID-19 outbreak, a new childhood multi-inflammatory syndrome temporally linked with SARS-CoV-2 infection has been reported worldwide. It has some similarities with Kawasaki disease (KD) and toxic shock syndrome (TSS) (1, 2). This condition has been named pediatric inflammatory multisystem syndrome temporally associated to SARS-CoV-2 infection (PIMS-TS) or multisystem inflammatory syndrome associated with coronavirus disease 2019 (MIS-C) (3–7). It typically displays 2–6 weeks after SARS-Cov-2 infection and has some overlapping features with KD, with a range of clinical presentations including mucocutaneous, respiratory, gastrointestinal, neurological, and cardiac symptoms. Like KD, no pathognomonic clinical findings or diagnostic tests exist. Unlike KD, however, MIS-C has been reported to predominantly affect adolescents and children aged older than 5 years and to be associated more frequently with cardiovascular (mainly myocarditis), and gastrointestinal involvement (1, 5, 6, 8, 9). The current definition of MIS-C, established by the World Health Organization in May 2020, refers to an individual aged 0–19 years, presenting with a fever for 3 days or longer, plus at least two of the following symptoms: rash; conjunctivitis; mucocutaneous inflammation; hypotension or shock; cardiac involvement; coagulopathy; and acute gastrointestinal symptoms. Laboratory evidence of increased inflammatory markers, such as erythrocyte sedimentation rate (ESR), C-reactive protein (CRP), or procalcitonin *plus* positive evidence of recent COVID-19 infection (by oronasal swab-PCR, serology, or antigen test) or likely recent contact, are required (10). While the underlying pathophysiology of MIS-C is currently under investigation, both the innate and adaptive immune response are thought to be strongly upregulated (11–13). One of the most intriguing theories hypothesizes that the SARS-CoV-2 spike protein can act as a “superantigen,” activating both T- and B-cells, leading to the hyperinflammatory state and a subsequent cytokine storm, similar to toxic shock syndrome (TSS) induced by the staphylococcal endotoxin B (14). Furthermore, it has been observed that patients affected by MIS-C develop vasculitis with endothelial damage, as shown by high levels of MCP-1 and VEGF-A, followed by an increase in pANCA (15). Among non-specific symptoms, severe complications including cardiovascular shock and multi-organ failure appear in most severe cases of MIS-C (16–18). Vasculitis and microthrombosis, particularly at the pulmonary level, were observed by necroscopy in some patients (19), but a case report of pulmonary vasculitis has never been documented. On the other hand, MIS-C shares some clinical features with other pediatric inflammatory multisystemic syndromes, such as KD, TSS, and macrophage activation syndrome (MAS), typically associated with endothelial damage and systemic vasculitis (20–22). Current practices and published guidelines for the treatment of MIS-C support the use of intravenous immunoglobulin (IVIG) and high-dose corticosteroids as the cornerstone of therapy (3, 4, 20, 23–28), in addition to antithrombotic prophylaxis. Treatment algorithms generally recommend biologic agents as second-line medication options after initial treatment with IVIG and steroids (22, 27, 29). Anakinra is a recombinant human interleukin-1 receptor antagonist that has been previously shown to be effective with limited side effects in patients with KD (29). Many studies have shown a similar efficacy of anakinra in severe cases of MIS-C, especially in patients with a clinical course complicated by severe myocarditis, shock, and a poor response to IVIG and steroid treatment (30–33). In two large cohorts, anakinra was successfully used in 24 of 186 and 8 of 183 patients, demonstrating an overt efficacy in cases of MIS-C with severe cardiac involvement (9, 34). We present a case of MIS-C complicated by pulmonary vasculitis successfully treated with sildenafil and high doses of anakinra.

## Case report

**Figure 1:**
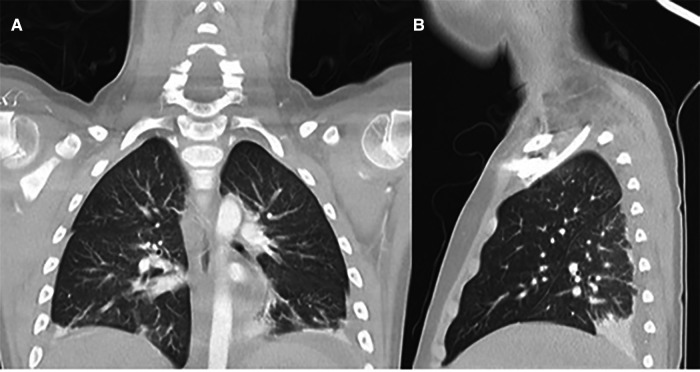
Chest computed tomography revealed multiple basal parenchymal thickening with small basal pleural effusion without signs of pulmonary embolism: (**A**) antero-posterior view; (**B**) latero-lateral view.

A previously healthy 7-year-old male patient was admitted to our Pediatric Department, complaining of unremitting fever above 39 °C for 5 days, fatigue, headache, and acute abdominal pain, unresponsive to non-steroidal anti-inflammatory drugs ,and antibiotic treatment with amoxicillin-clavulanate. At admission on physical examination, he presented with bilateral non-exudative conjunctivitis, skin rash on the trunk and upper limbs, and neck rigidity with some sign of meningism. He had had a pauci-symptomatic SARS-CoV-2 infection approximately 4 weeks before, characterized by 2 days of fever, asthenia, and headache. The infection had been ascertained by a positive oronasal swab for SARS-CoV-2. Laboratory findings showed marked elevation of CRP (275 mg/L) and ESR (66 mm/1^th ^h), but also of ferritin (556 mg/L) and brain natriuretic peptide (pro-BNP; 3408 pg/ml). Marked lymphopenia (440/mmc) and hypoalbuminemia (23 g/L) were also found at admission. A molecular swab for SARS-CoV-2 was negative. The work-up excluded Epstein Barr virus, cytomegalovirus, parvovirus B19, adenovirus, and HIV infection. The echocardiography performed when he presented to the pediatric emergency unit showed signs of endocarditis (mild aortic and mitral regurgitation) with normal contractility of the myocardium and normal pulmonary pressure (26 mmHg). The abdominal ultrasound showed a thin layer of fluid among the intestinal loops and the chest x-ray showed a homogeneous parenchymal thickening in the left paracardiac basal area with small left basal pleural effusion. In view of his medical history, clinical examination, and blood and instrumental tests, a diagnosis of MIS-C related to COVID-19 infection was made. According to guidelines, IVIG at a dose of 2 g/kg associated with a high dose of metilprednisolone at a dose of 30 mg/kg were started (Table 1). Subcutaneous thromboprophylaxis with low-molecular weight heparin (LMWH) at a dose of 100 UI/kg was also initiated (Table 1). After the third bolus of high-dose corticosteroids, his fever and neurological symptoms persisted, together with a persistent elevation of CRP (135 mg/L). Therefore, intravenous anakinra (a 2 mg/kg/dose every 6 h) was started while intravenous methylprednisolone (1 mg/kg b.i.d.) was continued (Tables 1 and 2), showing a rapid response, with the disappearance of the fever and skin rash and the reduction of CRP (60 mg/L) and pro-BNP (546 pg/ml). After 2 days, he suddenly presented with progressive dyspnea, increased labored breathing, and hypoxia (SatO_2_: 85% in ambient air room), without any other associated symptoms. The delivery of O_2_ by humidified high-flow nasal cannula (HFNC) was started with a FiO_2_ 50%. A chest CT scan was performed, showing multiple basal parenchymal thickening with small basal pleural effusion (Figure 1). An arterial blood gas analysis showed severe hypoxia (PaO_2_, 56 mmHg; PaO_2_/FiO_2_ ratio, 170 mmHg). The blood tests showed new slowly increasing inflammatory markers (CRP 113 mg/L, ESR 68 mm/1^th^ h). Because of the increased work of breathing and persistent altered gas exchange, the patient was transferred to the pediatric intensive care unit (PICU), and then put on invasive mechanical ventilation. Administration of anti-IL-1 was modified and a continuous infusion of high-dose anakinra (12 mg/kg/day) was started (Tables 1 and 2). Echocardiography revealed increased pulmonary pressure (40 mmHg) with normal heart ejection fraction (55%). These results confirmed the hypothesis of ongoing pulmonary vasculitis involving pulmonary arterioles. Therefore, intravenous sildenafil at a dose of 0.15 mg/kg/day was started (35, 36) (Table 1), with a progressive reduction of pulmonary pressure (23 mmHg) and resolution of the clinical picture of respiratory failure. After 36 h from the start of the treatment, the patient’s clinical condition as well as laboratory parameters remarkably improved, and he was extubated and again transferred to our department. The treatment was well tolerated and after 3 days the CRP fell in the normal range, sildenafil was stopped, and the biologic treatment was escalated, taking off one dose of anakinra every 3 days, up to discontinuation (Table 1). At discharge, echocardiography revealed signs of previous cardiac involvement with persistent mild mitral regurgitation and total regression of aortic regurgitation, and chest x-ray showed a resolution of parenchymal thickening and pleural effusion. The treatment with LMWH was shifted to oral cardioaspirin at a dose of 5 mg/kg/day (max 100 mg/day) (Table 1). After 2 months of follow-up, the clinical examination was normal, and the blood test and cardiologic assessment with echocardiography also normalized; therefore, the cardioaspirin therapy was stopped.

**Table 1:** Summary of the immunomodulatory and thromboprophylaxis therapy administered to our patient affected by MIS-C complicated with pulmonary vasculitis.

Day of admission	IVIG	Methylprednisolone (intravenous)	Prednisone (oral)	Anakinra (intravenous)	Sildenafil (intravenous)	LMWH (subcutaneous)	Aspirin (oral)	PICU
1	2 g/kg^a^	30 mg/kg^b^				100 UI/kg		
2		30 mg/kg^b^				100 UI/kg		
3		30 mg/kg^b^				100 UI/kg		
4		1 mg/kg BID		2 mg/kg QID		100 UI/kg		
5		1 mg/kg BID		2 mg/kg QID		100 UI/kg		Yes
6		1 mg/kg BID		12 mg/kg^c^	0.05 mg/kg TID	100 UI/kg		Yes
7		1 mg/kg BID		12 mg/kg^c^	0.05 mg/kg TID	100 UI/kg		Yes
8		1 mg/kg BID		12 mg/kg^c^	0.05 mg/kg TID	100 UI/kg		
9		1 mg/kg BID		2 mg/kg QID	0.05 mg/kg BID	100 UI/kg		
10		1 mg/kg BID		2 mg/kg QID	0.05 mg/kg/day	100 UI/kg		
11		1 mg/kg/day		2 mg/kg TID		100 UI/kg		
12		1 mg/kg/day		2 mg/kg TID		100 UI/kg		
13			1 mg/kg/day	2 mg/kg BID		100 UI/kg		
14			0.75 mg/kg/day	2 mg/kg BID		100 UI/kg		
15			0.75 mg/kg/day	2 mg/kg BID		100 UI/kg		
16			0.5 mg/kg/day	2 mg/kg/day		100 UI/kg		
17			0.5 mg/kg/day	2 mg/kg/day		100 UI/kg		
**Discharge**			0.25 mg/kg/day for other 2 days and then stop				5 mg/kg until 8 weeks from onset	

BID, Bis in die; TID, Ter in die; QID, Quarter in die.

^a^ (max80 g) to be administered over at least 12 h. In patients with heart failure immunoglobulins should be administered over at least 16 h. or, alternatively, the total dose should be split into two infusions 12 h apart.

^b^ in 3 h (max 1 gr).

^c^ Continuous infusion.

**Table 2:** Different modalities of Anakinra administration in MIS-C patients.

Intravenous		Subcutaneous
Pulse	Continuous infusion	
• Indication: in case of persistent disease activity 48 h after first-line treatment • In case of MAS. or shock with cardiac failure in adjunction to corticosteroids and IVIG • Dilute the vial of anakinra (100 mg) in 100 ml of 0.9% NaCL • Use bags (not plastic, PVC or glass containers) • Mix gently by inversion • Administer the diluted anakinra solution intravenously, *via* an infusion pump, in one hour, immediately after preparation, so as to administer 2 mg/kg (max 100 mg) per dose • Discard the remaining contents • It's possible to repeat the same preparation, every 6 h (4 times/day)	• Indication: in case of persistent disease activity 48 h after first-line treatment • In case of MAS or shock with cardiac failure in adjunction to corticosteroids and IVIG • Dilute the vial of anakinra (100 mg) in 100 ml of 0.9% NaCL • Use bags (not plastic, PVC or glass containers) • Mix gently by inversion • Administer the diluted anakinra solution intravenously, via an infusion pump, over 6 h, immediately after preparation, at dose of 2–3 mg/kg per dose (max 100 mg); discard the remaining contents • Repeat the same preparation, every 6 h, as a continuous infusion (4 times/day) • Maximum daily dose 12 mg/kg or 400 mg	• Indication: in case of persistent disease activity 48 h after first-line treatment • Administer anakinra with the pre-filled vial subcutaneously on the belly, thigh or arm area • At dose of 4–6 mg/kg/day (max 100 mg x dose)

This tble shows how to administer Anakinra in MIS-C patients. In view of acute hyperinflammatory disease, we advise intravenous use as a pulse or continuous infusion depending on the severity of the disease and organ involvement.

## Discussion

MIS-C is a post-infectious hyperinflammatory syndrome caused by a dysregulated immune response to SARS-CoV-2 infection with severe morbidity and mortality (15, 19–22). Diffuse vascular-endothelial damage is likely to play a major role in cases with severe cardiac and neurological involvement (15, 19–22). The strong association between COVID-19 and coagulopathy suggests that multiple molecular pathways are involved and dysregulated through the disease progression, contributing to the development of thrombosis; this is true also for MIS-C (37). Endothelial dysfunction and barrier disruption lead to immune cell infiltration, and proinflammatory cytokine production, as well as thrombosis (38). MIS-C seems to have specific effects on the coagulation profile leading to hypercoagulability and a thrombogenic state; in particular, complement activation has been hypothesized as a favoring factor in thrombosis development (39). Recent studies in children have pointed to an increase in endothelial dysfunction markers in MIS-C, with a rise in soluble C5b-9 (which represents the activated product of the terminal complement cascade) and altered red blood cell morphology (39, 40). In patients with MIS-C, high levels of fibrinogen and D-dimers increase the likelihood of a thrombotic state (37). As far as we know, our case represents the first report of pulmonary arterial hypertension (PAH) likely due to pulmonary vasculitis in a pediatric patient with MIS-C. We examined several explanations for the PAH in our young patient. First, we considered a pulmonary embolism, which was ruled out by the CT scan. In addition, the echocardiographic assessment was negative for underlying heart conditions or cardiac failure. Therefore, we considered the diagnosis of pulmonary vasculitis as the only possibility of the sudden dyspnea associated with an increase of PAH after the exclusion of cardiac and other pulmonary causes, as occurring in some cases of KD with pulmonary involvement (36). Therefore, we administered sildenafil, a type 5 phosphodiesterase inhibitor, known to induce vasodilation, particularly in the pulmonary arterial district, and to inhibit endothelial proliferation. This therapy has already been shown to be effective in treating children with PAH in different conditions (35). We believe that in our case the association of sildenafil with high doses of anakinra was decisive in preventing the progression of the disease. In this regard, according to different international societies of pediatrics and rheumatology, anakinra would be considered in MIS-C patients refractory to first-line medications (IVIG and corticosteroids), or in cases complicated by MAS or shock (3, 4, 23, 27, 33). However, the effective control of the hyperinflammatory condition can depend on a window of opportunity that the step-up approach does not always allow respect. We believe that in our case, the delay of diagnosis (admission to the hospital on the 5th day of fever) along with the decision of starting the therapy with IVIG and high-dose steroids alone, without anakinra, may have played a role in the suboptimal control of symptoms, paving the way to the development of pulmonary vasculitis requiring admission to the PICU. This decision was based on recommendations from guidelines to start anakinra in MIS-C in patients without cardiac failure or MAS only in case of uncontrolled disease at 48 h after first-line treatments (3, 4, 23, 27). However, neurological involvement with meningoencephalitis and pulmonary involvement with acute respiratory failure are determining elements for admission to the PICU (8, 9, 18). In our report, the worsening of the cardio-respiratory compromise was decisive for PICU admission and prompted us to introduce a second-line treatment with high-dose intravenous anakinra and sildenafil. In our opinion, the presence of neurological involvement with overt meningism signs should be considered as criteria for starting a more aggressive treatment with anakinra, using this drug as a first-line approach. In our case, such therapy on the second day of hospitalization, after the failure of the first day of IVIG and infusion of high-dose corticosteroids, could have been started. This would have made it possible to anticipate the use of anakinra by 48 h, probably within the window of opportunity to avoid the need for intensive care and the possible complications of the hyperinflammatory picture. However, sildenafil and high doses of intravenous anakinra treatments allowed the patient to have a short-term hospitalization in the PICU and a total normalization of the neurological, cardiological, and pulmonary picture and normal long-term outcome.

## Conclusion

As far as we know, our case represents the first report in pediatric literature of pulmonary vasculitis in MIS-C patients. One limitation of our report is that no pulmonary biopsy was performed to confirm our hypothesis of pulmonary vasculitis as a cause of severe respiratory distress and altered gas exchange requiring invasive mechanical ventilation. On the other hand, the rapid improvement registered in our patient with sildenafil and high doses of anakinra made the biopsy unethical to perform. We believe that the use of sildenafil and high-dose intravenous anakinra may represent an effective rescue therapy in severe cases of MIS-C with likely pulmonary vascular involvement, allowing a more rapid discharge from PICU and possibly improving the long-term outcome in these patients.

## Data Availability

The original contributions presented in the study are included in the article/Supplementary Material, further inquiries can be directed to the corresponding author/s.
